# Personalized Risk-Based Screening Design for Comparative Two-Arm Group Sequential Clinical Trials

**DOI:** 10.3390/jpm12030448

**Published:** 2022-03-12

**Authors:** Yeonhee Park

**Affiliations:** Department of Biostatistics and Medical Informatics, University of Wisconsin-Madison, Madison, WI 53705, USA; ypark56@wisc.edu

**Keywords:** adaptive randomization, Bayesian inference, clinical trials, personalized medicine, probit model, screening

## Abstract

Personalized medicine has been emerging to take into account individual variability in genes and environment. In the era of personalized medicine, it is critical to incorporate the patients’ characteristics and improve the clinical benefit for patients. The patients’ characteristics are incorporated in adaptive randomization to identify patients who are expected to get more benefit from the treatment and optimize the treatment allocation. However, it is challenging to control potential selection bias from using observed efficacy data and the effect of prognostic covariates in adaptive randomization. This paper proposes a personalized risk-based screening design using Bayesian covariate-adjusted response-adaptive randomization that compares the experimental screening method to a standard screening method based on indicators of having a disease. Personalized risk-based allocation probability is built for adaptive randomization, and Bayesian adaptive decision rules are calibrated to preserve error rates. A simulation study shows that the proposed design controls error rates and yields a much smaller number of failures and a larger number of patients allocated to a better intervention compared to existing randomized controlled trial designs. Therefore, the proposed design performs well for randomized controlled clinical trials under personalized medicine.

## 1. Introduction

Personalized medicine is a new paradigm motivated by the possibility that patients’ response to a particular treatment is heterogeneous, which may be due to biological covariates. Only a subset of patients is sensitive to, and benefit from, the treatment. Thus, a traditional one-size-fits-all remedy may not be the best option for some patients, even though the standard of care for a disease generally has a well-established track record. In the era of personalized medicine, molecularly targeted agents have been developed for disease treatment and prevention, e.g., trastuzumab [1,2], crizotinib [3,4], and erlotinib [5,6]. Novel statistical methods and clinical trial designs have been proposed for the novel targeted therapy. Park [7] reviewed statistical methods evaluating the effect of the targeted therapy with a certain genetic mutation on multiple disease types. Biomarker-based clinical trial designs have been proposed to address the one-size-fits-all issue [8,9,10,11]. Adaptive enrichment designs propose the enrichment rule to identify the patients who are expected to get more benefit from the experimental treatment and restrict the enrollment adaptively to the treatment sensitive patients [12,13]. In this paper, we are interested in how personalized medicine works on randomization of treatments for clinical trials.

Randomization is critical in clinical trials to remove any systematic bias for detecting the treatment effect and thus powerful to ensure validity in the comparative clinical trials. Most of randomized controlled trials use a fixed randomization to allocate participants to the treatments being compared, i.e., the allocation ratio 1:1 or 2:1 is commonly used in comparative two-arm clinical trials. The fixed randomization makes simple to execute the clinical trials. However, in the era of the personalized medicine, it would make investigators hesitant to assign equal number of patients to each treatment if the trial enrolls patients regardless of enrollment restriction to a targeted subgroup based on empirical evidence of the efficacy of the treatments. As an effective approach to address the ethical problem, adaptive randomization assigns more future patients to the better performing treatment based on the accumulating information on patients’ response to the treatments. Using the skewed allocation probability, response-adaptive randomization (RAR) designs for binary response trials have been proposed [14,15,16,17]. The optimal allocation probability to treatments was proposed in that sample size is minimized [18] or total number of failures is minimized [19,20]. To incorporate patients’ covariate information in RAR designs, the response probability conditioning on the covariates is estimated for RAR [17,21,22,23,24].

In this paper, we propose a personalized risk-based screening design for comparative two-arm group sequential clinical trials. The proposed design follows the group sequential manner with the first look used for a burn-in stage. It collects some preliminary data to facilitate the regression fitting and adaptive decision of the intervention assignment for the next stages. We propose personalized randomization using a Bayesian covariate-adjusted response-adaptive randomization based on adaptive regression of response on informative covariates to randomize a patient with the given vector of covariates to the intervention from which the patient is expected to get more benefit based on the accumulating information. Using risk factors to build the personalized risk-based allocation probability, the design provides individually tailored randomization of screening modality. Moreover, we propose a group sequential test in personalized allocation and Bayesian monitoring rule to compare screening effects and maintain the error rates.

The rest of this paper is organized as follows. In Section 2, we describe a motivating trial for cancer screening and propose a design structure, probability model, and methods for the personalized screening trial design. In Section 3, we evaluate the operating characteristics of the proposed design using simulation studies. We provide discussion in Section 4.

## 2. Personalized Risk-Based Screening Design

### 2.1. Motivating Trial

Tomosynthesis Mammographic Imaging Screening Trial (TMIST) is a Phase III trial study, which starts on July 2017 and will be completed by August 2030 (The study identifier is NCT03233191). TMIST randomizes women between the ages of 45 and 74 to either tomosynthesis mammography (3D mammography) or standard digital mammography (2D mammography) with equal probability and evaluates the mammographic accuracy for breast cancer screening. The primary endpoint of the study is the incidence of advanced breast cancer, and the trial was designed to compare the proportion of women diagnosed with an advanced breast cancer between two screening modalities. In an era of personalized medicine, it is essential to develop methods and trial designs for personalized risk-based screening using breast density, tumor subtyping, and genomics [25].

### 2.2. Design Structure

Motivated by TMIST considering two screening disparities, digital breast tomosynthesis mammography and standard digital mammography, we consider a comparative group sequential clinical trial with patients individually randomized to experimental treatment *A* or control *B* based on accumulating data.

**Figure 1:**
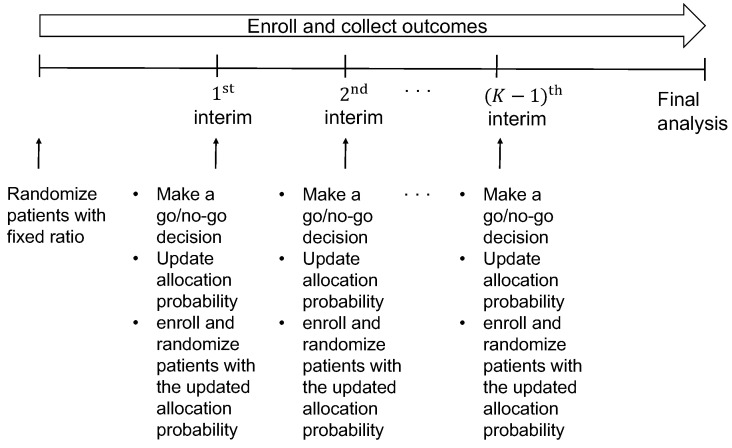
Schema of the proposed design.

Our design enrolls a maximum of *N* patients sequentially in cohorts of sizes $n_{1},\ldots ,n_{K}$ with $N=\sum_{k=1}^{K}n_{k}$. The design uses a Bayesian group sequential monitoring, described in Section 2.5 below, for superiority or futility at interims to compare *A* to *B* in the adaptively randomized patients. The schema of the design is shown in Figure 1. The trial begins by enrolling patients according to the eligibility criteria for the first cohort of $n_{1}$ patients. It randomizes the patients to *A* or *B* with equal probability. When the $n_{1}$ patients have been enrolled and their outcomes are available, the superiority or futility of the experimental treatment *A* against the control *B* is monitored at the first interim. If the monitoring shows that *A* is superior or futile, the trial is terminated. However, if the trial is not stopped early, then we fit the regression model of response on a vector of patients’ characteristics and treatment to estimate the personalized allocation probability, given in (2) in Section 2.4 below. The allocation probability is updated to randomize the treatment adaptively and individually for the next enrollment of the second cohort. This procedure is repeated until the end of the trial. If the maximum sample size *N* is reached and the last patient’s outcome has been evaluated, a final analysis is performed.

### 2.3. Probability Model

Let *G* be an indicator of treatment group taking 1 for receiving experimental treatment *A* and 0 for receiving control *B*. Let *Y* be a binary indicator of events, e.g., deaths. For each patient, we assume that a vector of informative covariates x is available at enrollment.

We describe a probability distribution for *Y* assuming a probit regression model

(1) $$\mathrm{Pr}\left(Y=1|G,x\right)=\Phi \left({\tilde{x}}^{⊤}\beta +G{\tilde{x}}^{⊤}\gamma \right),$$

where Φ(·) denotes the cumulative distribution function of standard normal variable, $\tilde{x}={\left(1,x^{⊤}\right)}^{⊤}$ and $\theta \equiv {\left(\beta^{⊤},\gamma^{⊤}\right)}^{⊤}$ denotes the regression coefficient parameter vector. Specifically, β is the vector of covariate main effects and γ is the vector of interaction effects between treatments and covariates including the main experimental versus control effect. Back to the motivating trial, *Y* is the indicator of having breast cancer. The probability in (1) indicates the chance of having an advanced breast cancer for the given screening method *G* and a vector of patients’ characteristics x. To interpret the breast cancer risk for screening, electronic health record, breast density, age, tumor subtyping, first-degree breast cancer family history, and genomics are candidates of the predictive covariates in the risk prediction model [26,27,28,29,30,31].

Assigning β and γ normal priors, the parameters are estimated by Bayesian inference. We used LearnBayes R package to fit Bayesian probit regression model.

### 2.4. Personalized Allocation for Adaptive Randomization

For each k=1,…,K−1, let $D_{k}$ be an accumulating data at the *k*th interim, i.e., a set of Y,G,x over the *k* cohorts. Let $p_{A}\left(x\right)=\mathrm{Pr}\left(Y=1|G=1,x\right)$, and $p_{B}\left(x\right)=\mathrm{Pr}\left(Y=1|G=0,x\right)$. Then, $p_{A}\left(x\right)-p_{B}\left(x\right)=\Phi \left({\tilde{x}}^{⊤}\beta +{\tilde{x}}^{⊤}\gamma \right)-\Phi \left({\tilde{x}}^{⊤}\beta \right)$, which is a function of unknown parameter $\theta ={\left(\beta^{⊤},\gamma^{⊤}\right)}^{⊤}$. To assign more patients to the better performing personalized treatment, we are interested in quantifying a likelihood of a patient with x benefiting more from the treatment *A* than *B*, i.e., $p_{A}\left(x\right)-p_{B}\left(x\right)<0$. Let $p_{k-1}\left(x\right)=\mathrm{Pr}\left(p_{A}\left(x\right)<p_{B}\left(x\right)|D_{k-1}\right)$ denote the posterior probability that a patient with covariates x is less likely to have an event under treating *A* than treating *B* based on accumulating data $D_{k-1}$. Assuming normal prior on θ for Bayesian probit regression model in Section 2.3, samples of θ are generated from the posterior distribution

$$\mathrm{Pr}\left(\theta |\mathrm{data}\right)=\frac{\mathrm{lik}(\mathrm{data}|\theta )\mathrm{prior}(\theta )}{\int \mathrm{lik}(\mathrm{data}|\theta )\mathrm{prior}(\theta )\;d\theta }\;$$

where lik(data|θ) denotes the likelihood function and prior(θ) denotes the prior distribution of parameter θ, and the posterior probability $p_{k-1}\left(x\right)$ is calculated. We provide how to compute the posterior probability $p_{k-1}\left(x\right)$ in Appendix A. The posterior probability is to reflect the personalized medicine, and patients’ characteristics x are incorporated into the posterior probability $\mathrm{Pr}(p_{A}<p_{B}|D_{k-1})$ used in Bayesian adaptive randomization [32] Then, we define the probability of randomizing a patient with covariates x in the *k*th cohort to the treatment *A* as

(2) $$\pi_{k,A}\left(x\right)=\frac{\sqrt{p_{k-1}\left(x\right)}}{\sqrt{p_{k-1}\left(x\right)}+\sqrt{1-p_{k-1}\left(x\right)}}.$$

This is an option considering the personalized allocation probability, which is a type of covariate-adjusted response adaptive randomization (CARA). To emphasize in the randomization ratio that patients can respond differently to the treatments, we prefer what we call personalized randomization over CARA. We use this allocation probability (2) for the proposed design to perform personalized randomization.

Alternative option is to consider another type of CARA given by

(3) $$\pi_{k,A}\left(x\right)=\frac{\sqrt{1-p_{k-1,A}\left(x\right)}}{\sqrt{1-p_{k-1,A}\left(x\right)}+\sqrt{1-p_{k-1,B}\left(x\right)}}$$

where $p_{k-1,A}\left(x\right)=\mathrm{Pr}\left(Y=1|G=1,x,D_{k-1}\right)$ and $p_{k-1,B}\left(x\right)=\mathrm{Pr}\left(Y=1|G=0,x,D_{k-1}\right)$. The personalized allocation probability (3) uses the estimated response rates of treatment *A* and *B* denoted by $p_{k-1,A}\left(x\right)$ and $p_{k-1,B}\left(x\right)$, which are obtained by posterior mean of parameter. In our motivating screening trial, the response is an event such as death. To build the personalized allocation probability which is skewed to patients who get more benefit, the allocation probability is proportional to $1-p_{k-1,\cdot }\left(x\right)=\mathrm{Pr}\left(Y=0|G,x,D_{k-1}\right)$ instead of $\mathrm{Pr}(Y=1|G,x,D_{k-1})$. This is the modified version using Bayesian inference from optimal allocation probability suggested by Rosenberger et al. [19].

The personalized allocation probabilities (2) and (3) are updated throughout the clinical trials based on the accumulating data. They change the treatment allocation probability and adaptively randomize more patients to the treatment arm that is superior according to the patients’ characteristics. Back to the motivating trial, using the risk predictive model in Section 2.3, we are able to perform data-driven personalized randomization. It builds the personalized risk-based allocation probability and randomizes more patients to the superior screening modality individually. The personalized randomization makes more reasonable in ethics and help clinicians and clinical trialists get more out of randomized clinical trials.

### 2.5. Group Sequential Test in Personalized Randomization

To effectively use the personalized randomizationin group sequential designs allowing early stopping, it is critical to preserve the overall type I error rate. As the response adaptive randomization (RAR) including CARA is considered based on the observed data, potential selection bias can occur. Moreover, the bias would be more serious if CARA is used when there exists an effect of informative covariates. Park [33] shows that group sequential designs using CARA are influenced by prognostic covariates and the overall type I error rate is not controlled. To address the issue of type I error rate inflation from using the personalized allocation and accommodate the possible change in eligibility of patients during the trial, it is required to propose an elaborate test statistic which preserves the error rates.

At the *k*th analysis, the trial enrolls patients of *k* cohorts sequentially. Based on the accumulated data $D_{k}$ from *k* successive cohorts, which might consist of *k* heterogeneous cohorts, the *k*th interim monitoring determines go or no-go of the trial. Let $\Delta_{k}$ be an expected subgroup-averaged treatment effect based on the *k*th cohort. Assuming that x determines the subgroups, we suppose that there are $I_{k}$ subgroups in the *k*th cohort denoted by $S_{i},\;i=1,\ldots ,I_{k}$. In the case where x is continuous, the dichotomization can be considered to define the subgroups, e.g., young and old groups for the age variable. A comparative treatment effect of the *k*th cohort is obtained by

(4) $$\Delta_{k}=\sum_{i=1}^{I_{k}}\left\{p_{A}\left(x\right)-p_{B}\left(x\right)\right\}\mathrm{Pr}\left(x\right)I\left(x\in S_{i}\right),$$

where I(·) denotes the indicator function. It is a function of parameter θ and indicates the expected difference of the response probability with respect to x over the kth cohort. Then, a group sequential test statistic is proposed as the weighted sum of the comparative treatment effect based on *k* cohorts, i.e.,

(5) $$T_{k}=\frac{\sum_{j=1}^{k}n_{j}\Delta_{j}}{\sum_{j=1}^{k}n_{j}}.$$

As the comparative treatment effect $\Delta_{k}$ is calculated by marginalizing the difference of response probability with respect to x, the test statistic $T_{k}$ does not indicate the treatment effect of the individual patient. It indicates the overall treatment effect based on the accumulating data at the *k*th analysis.

When there are a few covariates, all possible combinations of subgroups are considered to obtain the comparative treatment effect $\Delta_{k}$. However, with more covariates, to avoid any computational burden or complexity, we suggest identifying the covariates whose main effect is significant so that they determine the subgroups in the *k*th cohort for the calculation of $\Delta_{k}$.

Let $\delta_{1}$ denote the minimal improvement for the experimental treatment to be deemed superior to the control and $\delta_{2}$ denote the minimal improvement so that the experimental treatment is considered worthy of further investigation. The values of $\delta_{1}$ and $\delta_{2}$ are pre-specified by clinicians or the study hypothesis. Let $\epsilon_{i}$, i=1,2,3 be the pre-specified probability cutoffs for superiority and futility monitoring rule. They are design parameters obtained by preliminary simulation-based calibration, where $\epsilon_{1}$ and $\epsilon_{3}$ control type I error rate α and $\epsilon_{2}$ controls type II error rate β. To save several rounds of calibrations, the initial cutoff values of $\epsilon_{1}$ and $\epsilon_{3}$ were selected as one minus target type I error rate, and the initial cutoff of $\epsilon_{2}$ was selected as one minus target type II error rate. To make sense with experts’ experience and knowledge, the survey results can be used to determine the level of evidence and calibrate for the monitoring rules [34]. If the type I error rate is lower/higher than the desirable level, we decrease/increase the value of $\epsilon_{1}$ and $\epsilon_{3}$, and if the calculated type II error rate is lower/higher than the desirable level, we decrease/increase the value of $\epsilon_{2}$. We repeat this calibration process until the desirable type I and II error rates are obtained. Then, the calibration procedure determines the cutoffs carefully to adjust the multiplicity of testing repeatedly over time and thus maintain the overall type I and II error rates at the nominal levels. It is widely used in Bayesian sequential designs [13,35,36,37]. Shi and Yin [38] provides the unified framework for the calibration procedure to search the cutoffs effectively.

Then, the Bayesian sequential monitoring rule is described as follows.

- At each interim k=1,…,K−1, the trial is terminated for superiority if $\mathrm{Pr}\left(T_{k}<\delta_{1}|D_{k}\right)>\epsilon_{1}$, or the trial is terminated for futility if $\mathrm{Pr}\left(T_{k}>\delta_{2}|D_{k}\right)>\epsilon_{2}$.
- When k=K (i.e., at final analysis), we argue that *A* is superior to *B* if $\mathrm{Pr}\left(T_{K}<\delta_{1}|D_{K}\right)>\epsilon_{3}$, and otherwise, *A* is not superior to *B*.

The posterior probabilities $\mathrm{Pr}(T_{k}<\delta_{1}|D_{k})$ and $\mathrm{Pr}(T_{k}>\delta_{2}|D_{k})$ are comupted by Bayesian inference (see Appendix A). The values of $\delta_{1}$ and $\delta_{2}$ are not necessarily to be the same in the decision rules. The proposed rule allows unequal values of $\delta_{1}$ and $\delta_{2}$ to increase the flexibility of the study.

## 3. Simulation Study

We assumed maximum sample size 210, which yielded 80% power to detect a response rate of 0.3 versus a null response rate of 0.5 based on a two-sample t-test with one-sided significance level α=0.05 under the traditional randomized clinical trial using the fixed equal randomization. Each patient was randomized to either experimental treatment *A* or control *B*. Two interim analyses were performed when the first 70 and 140 enrolled patients completed the evaluation of the response. At interims, we monitored the superiority or futility of the treatment *A* against *B*. A final analysis was performed after the last patient completed follow-up to argue the experimental treatment *A* is superior to *B*.

In the following, we first identified the challenging issues in personalized allocation based on the conventional group sequential test. Next, we investigated the performance of the proposed design and verified if the issues are addressed.

### 3.1. Type I Error Rate Inflation

We considered four group sequential clinical trial designs: traditional randomization with 1:1 (Trad), response-adaptive randomization without incorporating covariates (RAR), and covariate-adjusted response-adaptive randomization using (2) and (3) (CARA1 and CARA2, respectively). For all designs, we used the fixed equal randomization for the first cohort of 70 patients but changed the randomization scheme at the first interim according to the design. Trad kept the fixed equal randomization throughout the trial, but other designs updated the allocation probability at each interim to randomize the patients for the next cohorts. RAR used the allocation probability which Rosenberger et al. [19] proposes. CARA1 and CARA2 used the personalized allocation probability described in (2) and (3), respectively. To make comparable, four designs performed the conventional group sequential test based on a chi-square test. We set the overall type I error rate to 0.05 for the group sequential test. The O’Brien–Fleming alpha spending function was used to specify the stopping boundaries for the sequential test in Trad, RAR, CARA1, and CARA2. To estimate the personalized allocation probability in CARA1 and CARA2, we fitted the Bayesian probit regression model assuming normal priors with the mean vector of the maximum likelihood estimate and diagonal covariance matrix with diagonal elements 4. The choice of prior was to avoid using the vague prior and help the error rates less inflated [33]. When we implemented the Bayesian inference, we ran 10,000 iterations and discarded the first 5000 iterations as burn-in.

We considered two binary covariates $x=(x_{1},x_{2})$ which were generated from a Bernoulli distribution with response probability 0.5. There were four possible subgroups of patients determined by the two covariates, i.e., patients with x=(1,1),(1,0),(0,1), or (0,0). Then, the response *Y* was generated from a Bernoulli distribution with the probability

(6) $$\mathrm{Pr}\left(Y=1|G,x\right)=\Phi \left(\beta_{0}+\beta_{1}x_{1}+\beta_{2}x_{2}+\gamma_{0}G+\gamma_{1}Gx_{1}+\gamma_{2}Gx_{2}\right).$$

We considered twenty scenarios, and the true parameters generating response in (6) were described in Table 1.

Table 1 provides the summary of the response rates $p_{A}=\mathrm{Pr}\left(Y=1|G=1,x\right)$ and $p_{B}=\mathrm{Pr}\left(Y=1|G=0,x\right)$ for the overall group and four subgroups. Scenarios 1–9 describe null scenarios where both an experimental treatment *A* and the control *B* have no difference in the response. Scenarios 10–20 describe alternative scenarios where the main experimental versus control effect exists. Scenario 1 shows the same response for *A* and *B* as 0.5 regardless of patients’ characteristics or treatment assignment, i.e., it has no main effect of covariates or the main experimental versus control effect. Scenarios 2 and 3 have the main effect of the first covariate (i.e., $\beta_{1}\neq 0$), while Scenarios 4 and 5 have the main effect of the second covariates (i.e., $\beta_{2}\neq 0$). Scenarios 6–9 have nonzero coefficients $\beta_{1}$ and $\beta_{2}$ implying that two covariates $x_{1}$ and $x_{2}$ have the main effect on the response. Thus, in Scenarios 2–9, the response rate depends on the covariates but does not depend on the treatment assignment. They indicate the cases where there is an effect of prognostic covariates. Scenario 10 does not have any effects of covariates but has the main experimental versus control effect, and it is the case the experimental treatment *A* has better efficacy in response (i.e., smaller response) than the control *B*. Compared to Scenarios 10–12 we consider the additional effect of prognostic covariate $x_{1}$ (i.e., $\beta_{1}\neq 0$) to the main experimental versus control effect, while Scenarios 13 and 14 consider the additional effect of predictive covariate $x_{1}$ (i.e., $\gamma_{1}\neq 0$) to the main experimental versus control effect. Furthermore, in Scenario 15, the first covariate $x_{1}$ has both prognostic and predictive effects. Scenarios 16 and 17 have an effect of prognostic covariate $x_{2}$ and the effect of treatment assignment. In Scenario 18, the second covariate $x_{2}$ has both prognostic and predictive effects. In Scenarios 19 and 20, both $x_{1}$ and $x_{2}$ have prognostic and predictive effects. Depending on the effects of prognostic or predictive covariates, in Scenarios 10–20, particular subgroups with the covariate profile are more likely to get benefit from one of the treatments than the other treatment. To better understand the subgroups of the covariate profile, we call *A* (or *B*)-sensitive patients if the patients with the covariate profile x are expected to respond better to *A* (or *B*) but not respond to *B* (or *A*). The better treatment for *A*-sensitive patients is *A*, and the better treatment for *B*-sensitive patients is *B*. For example, in Scenario 14, patients with $x_{1}=0$ are *A*-sensitive; in Scenario 18, patients with $x_{2}=0$ are *A*-sensitive; in scenario 19, patients except x=(1,0) are *A*-sensitive; and in scenario 20, patients with x=(1,1) are *B*-sensitive and patients with x=(0,0) are *A*-sensitive.

Table 2 shows the estimated rejection probability to detect the difference of the response rate between treatments *A* and *B* based on 1000 simulated trials. The rejection probability under the null scenarios (i.e., Scenarios 1–9) indicates the overall type I error rate, and the rejection probability under the alternative scenarios (i.e., Scenarios 10–20) indicates the power. Trad and RAR preserved the type I error rate at the target level of 0.05 for all null scenarios. In addition, CARA2 worked well to control the overall type I error rate except for Scenarios 5 and 7. Specifically, under CARA2 using the personalized allocation probability (3), the estimated type I error rates were inflated at 10–17% in Scenarios 5 and 7. However, CARA1 failed in most null scenarios when there exists an effect of the prognostic covariate(s). Specifically, CARA1 using the personalized allocation probability (2) led to serious error inflation at 25–40% by the prognostic covariates in Scenarios 5 and 7. To investigate the type I error rate inflation in Scenarios 5 and 7, we looked at the distribution of the subgroups in each treatment arm *A* or *B* for all designs. The mean and standard deviation of the allocation probability of the treatment for each subgroup are reported in Table 3. We observed that designs using personalized randomization, e.g., CARA1, CARA2, and BaCARA, led to the large variability of the distributions compared to Trad and RAR which controlled the overall type I error rate. Under CARA1 and CARA2, the conventional group sequential test did not work properly in the presence of the effect of prognostic covariate(s), and we observed large inflations of overall type I error rate. However, under BaCARA, the overall type I error rates were less likely to be inflated, which resulted from the proposed group sequential test statistics considering the differences in treatment effect within subgroups. Depending on the difference in the response rate for each covariate profile and the prevalence of the subgroups, the outcomes were influenced by the covariates.

**Figure 2:**
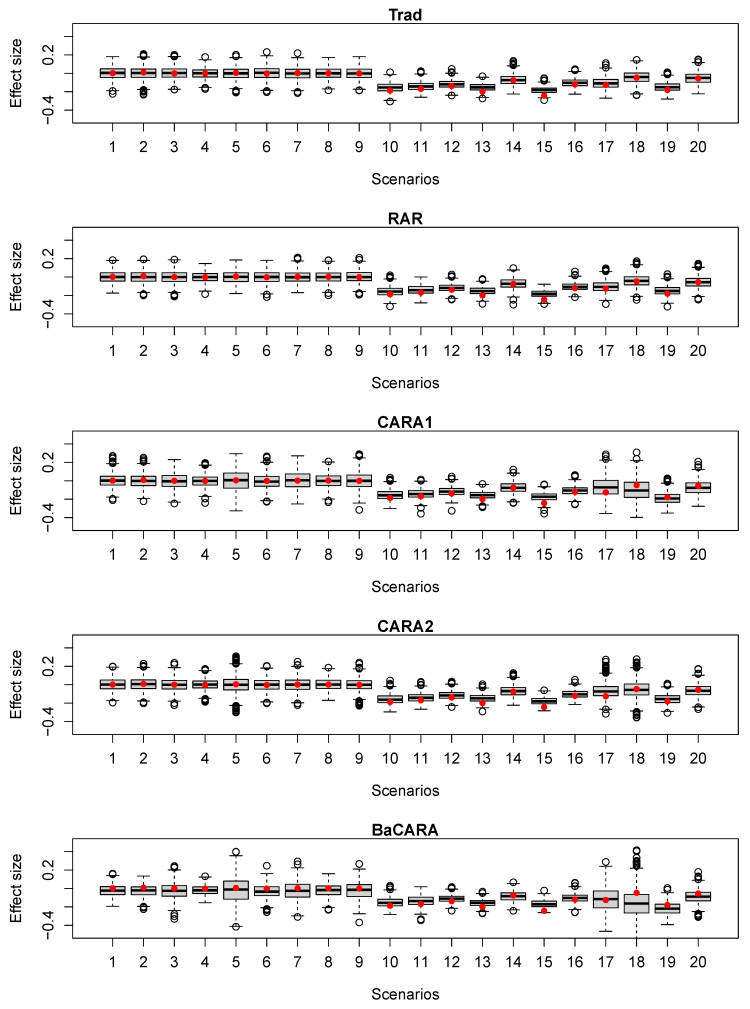
Boxplots of the estimated difference in the response probability between *A* and *B* (i.e., effect size) at final analysis. The red dots indicate the true effect sizes of the scenarios.

Under the alternative scenarios, Trad yielded a power which ranged from 0.10 to 0.98 depending on the overall difference between $p_{A}$ and $p_{B}$. As the power 80% was justified by the difference of 0.2 from the response probability of $p_{B}=0.5$, the power for each scenario varied according to the smaller or larger treatment effect difference and the null response probability $p_{B}$. RAR generally yielded similar or a little smaller power than Trad. CARA2 showed similar or larger power compared to Trad and RAR in most scenarios (except for Scenario 17). In most scenarios where the treatment effect difference or subgroup effect difference was less than 0.2 (i.e., Scenarios 10–16), CARA1 yielded similar or smaller power compared to Trad and RAR. However, when the treatment effect difference or subgroup effect difference became larger (i.e., in Scenarios 18–20), CARA1 led to much larger power than Trad and RAR. We also provided boxplots of the estimated difference between $p_{A}$ and $p_{B}$ at the final analysis for all designs in Figure 2. Therefore, CARA1 was more sensitive to the prognostic covariates than CARA2 and was more likely to inflate the error rates.

Table 4 shows other operating characteristics of the designs such as the average difference of the number of patients assigned to *A* and *B* and the average number of failures (i.e., events) across 1000 simulated trials. Compared to Trad, RAR and CARA change the allocation ratio and randomize more patients to the superior treatment. Under Trad, the averaged difference of the number of patients assigned to *A* and *B* ranged from −1.176 to 0.924 with an average of 0.065 across the alternative scenarios. Under RAR, the averaged difference of the number of patients assigned to *A* and *B* ranged from 0.482 to 3.082 with an average of 2.023 across the alternative scenarios. Under CARA1, the averaged difference of the number of patients assigned to *A* and *B* ranged from 16.808 to 48.808 with an average of 36.466 across the alternative scenarios. Under CARA2, the averaged difference of the number of patients assigned to *A* and *B* ranged from 0.316 to 17.598 with an average of 6.654 across the alternative scenarios. CARA1 and CARA2 showed a larger number of patients assigned to the superior treatment *A* than Trad and RAR. The gain was much larger when CARA1 is considered, which was resulted from the effective use of the personalized allocation probability based on the accumulating data. It also resulted in a smaller number of failures under CARA1 than other designs. Under Trad, the number of failures ranged from 33.47 to 135.25 with an average of 70.03; under RAR, the number of failures ranged from 33.21 to 134.73 with an average of 69.92; and under CARA2, the number of failures ranged from 32.73 to 133.56 with an average of 69.29. All three designs showed similar performance in the number of failures, i.e., CARA1 did not show apparent gain in the number of failures. However, under CARA1, the number of failures ranged from 30.12 to 130.58 with an average of 64.60.

The simulation study tells us that effective use of the personalized allocation probability can lead to the inflation of the overall type I error rate but is more ethical by assigning more patients to the superior treatment and yields a smaller number of failures. Therefore, it is critical to maintain the overall type I error rate in personalized allocation and improve clinical benefit while inheriting the advantages of CARA designs.

### 3.2. Evaluation of the Proposed Design: Preservation of Type I Error Rate

We observed the inflation of the overall type I error rate using CARA1 in Table 2, which came from the prognostic covariates’ effect and sequential personalized allocation. Using a conventional group sequential test to detect the overall treatment difference in the response probability did not work well when the randomization depended on patients’ characteristics. Patients receiving a certain treatment might not be homogeneous, and they responded differently to the treatment. To accommodate this heterogeneity and control the type I error rate, the group sequential test in personalized allocation was proposed in Section 2.5.

For convenience, we called the proposed design BaCARA, which used the personalized allocation probability (2) to randomize the patients and monitor the treatment effect based on the proposed group sequential test statistic (5) through the Bayesian sequential monitoring rule. We evaluated the operating characteristics of BaCARA through simulations. We followed the same simulation settings as in Table 2, Table 3 and Table 4. To compare the results with Trad, RAR, CARA1, and CARA2, we included the results of BaCARA in the last column of Table 2, Table 3 and Table 4. Assuming the minimal improvements $\delta_{1}=\delta_{2}=0$, we calibrated $\epsilon_{1}=0.995$, $\epsilon_{2}=0.75$, and $\epsilon_{3}=0.98$ by preliminary simulations to control the error rates under the null scenarios (i.e., Scenarios 1–9 in Table 1) and the alternative scenario with $p_{A}=0.3$ and $p_{B}=0.5$ (i.e., scenario 10 in Table 1).

We observed in Table 2 that BaCARA preserved the overall type I error rate at the target level of 0.05, implying that the inflation issue of CARA1 was addressed. Compared with Trad and RAR designs, under BaCARA, the overall type II error rates seemed to be controlled well (i.e., in Scenarios 10–20). Similar to CARA1 and CARA2, BaCARA was more powerful than Trad and RAR in Scenarios 18–20 where patients’ response to the treatment was more heterogeneous, i.e., the treatment effect difference or subgroup effect difference was relatively larger. In addition, in Scenario 17 where CARA2 yielded a large inflation of type II error rate, BaCARA showed a large power compared to other designs. Thus, BaCARA improved the performance of CARA1 and CARA2 using the personalized allocation probability in that it preserved the overall type I and II error rates. BaCARA was appropriate to use for group sequential clinical trials incorporating patients’ characteristics into the adaptive randomization.

In Table 4, BaCARA showed better performance in the difference of the number of patients assigned *A* and *B* than Trad, RAR, and CARA2, but it had smaller differences of the number of patients assigned *A* and *B* than CARA1 in most scenarios. Under BaCARA, the difference of the number of patients assigned *A* and *B* ranged from 17.718 to 33.606 with an average of 26.634. However, BaCARA yielded a smaller number of failures across scenarios than Trad, RAR, CARA1, and CARA2. Under BaCARA, the number of failures ranged from 25.82 to 116.36 with an average of 57.84. Such an improvement came from the effective group sequential test as well as the personalized allocation. The proposed design led to the improvement of clinical benefit and provided a better suggestion to effectively use personalized randomization for personalized medicine.

## 4. Discussion

We proposed a personalized risk-based screening design using Bayesian covariate-adjusted response-adaptive randomization for comparative two-arm clinical trials. Following the group sequential procedure, we adaptively built the personalized allocation probability using the risk factors to randomize more patients to the most desirable individualized intervention and minimize the number of events. We also proposed a new group sequential test to address the challenging issues in the personalized allocation. The proposed Bayesian monitoring rule determined go or no-go of the trial at interims based on accumulating data, and the proposed design preserved the type I error rate through the calibrated cutoffs for the Bayesian monitoring rule.

We compared the performance of the proposed design to the randomized controlled trial designs such as traditional, RAR, and CARA designs. Even though RAR design assigned more patients to the better performing intervention and thus was ethical compared to the traditional randomized controlled trial design, the expected number of failures was not different, and the improvement of clinical benefit was not clear. In addition, in RAR, all eligible patients were enrolled and randomized without any restriction considering patients’ characteristics, which was not appropriate in personalized medicine. By incorporating patients’ characteristics into randomization, CARA design led to a larger allocation of patients to the better performing intervention than RAR design. However, CARA designs could be sensitive to the prognostic covariate effect and inflate the overall type I error rate. Furthermore, in our simulations, it was not clear to achieve a significant improvement in clinical benefit (i.e., the smaller number of events) compared to the traditional and RAR designs. Taking all of the above into account, the proposed design was the most appropriate to use for two-arm personalized screening clinical trials.

The proposed design is flexible and extended to the followings. First, assuming that informative covariates are not specified at the beginning of the trial, covariate selection methods can be carried out in the burn-in stage. The selected covariates with the significant effect are used in the remaining stages to randomize and test the screening effect. Second, our Bayesian sequential monitoring rule is flexible and can be modified according to the study objectives. For example, additional monitoring rules based on surrogate or safety endpoint can be included to make a data-driven decision throughout the trials. This also allows us to learn health systems along with the trials. Third, personalized randomization can be generalized for multi-arm trials, and each arm is compared to the control using the proposed test. For example, to calculate the allocation probability (2) of randomizing a patient with x to the treatment *A*, the posterior probability of $p_{A}\left(x\right)<p_{B}\left(x\right)$ for comparing with the control *B* is replaced with the posterior probability that the treatment *A* offers the minimum response rates among all treatment arms [39]. Villar et al. [40], Ryan et al. [41], and Viele et al. [42] provide some directions under consideration for the multi-arm trials.

## Figures and Tables

**Table 1:** Simulation scenarios: True model parameters when $x_{1}$ and $x_{2}$ are independently generated from a Bernoulli distribution with response probability 0.5. Note that “sc” denotes scenarios.

sc.	$\beta_{0}$	$\beta_{1}$	$\beta_{2}$	$\gamma_{0}$	$\gamma_{1}$	$\gamma_{2}$	Overall		x=(1,1)		x=(1,0)		x=(0,1)		x=(0,0)	
							$p_{A}$	$p_{B}$	$p_{A}$	$p_{B}$	$p_{A}$	$p_{B}$	$p_{A}$	$p_{B}$	$p_{A}$	$p_{B}$
1	0	0	0	0	0	0	0.500	0.498	0.496	0.493	0.503	0.502	0.500	0.500	0.499	0.498
2	−0.5	0.5	0	0	0	0	0.407	0.398	0.502	0.488	0.497	0.499	0.318	0.302	0.310	0.296
3	−0.5	1	0	0	0	0	0.499	0.499	0.695	0.689	0.691	0.694	0.313	0.300	0.299	0.309
4	−1	0	0.5	0	0	0	0.231	0.233	0.302	0.311	0.157	0.155	0.312	0.312	0.155	0.157
5	−1	0	2	0	0	0	0.501	0.496	0.845	0.836	0.159	0.157	0.844	0.838	0.156	0.152
6	−0.5	0.5	0.5	0	0	0	0.498	0.500	0.689	0.696	0.499	0.503	0.500	0.493	0.303	0.308
7	−0.5	1	1	0	0	0	0.658	0.656	0.932	0.931	0.695	0.698	0.688	0.692	0.314	0.305
8	−0.5	−0.5	0.5	0	0	0	0.321	0.319	0.312	0.304	0.159	0.159	0.500	0.497	0.303	0.312
9	−0.5	−1	1	0	0	0	0.344	0.345	0.313	0.309	0.069	0.070	0.699	0.695	0.300	0.312
10	0	0	0	−0.5	0	0	0.312	0.499	0.310	0.505	0.311	0.501	0.315	0.497	0.309	0.491
11	−0.5	0.5	0	−0.5	0	0	0.235	0.404	0.304	0.503	0.314	0.501	0.158	0.308	0.158	0.305
12	−0.5	−0.2	0	−0.5	0	0	0.138	0.276	0.114	0.242	0.115	0.240	0.158	0.307	0.163	0.317
13	−0.5	0	0	−0.5	−0.5	0	0.112	0.311	0.067	0.315	0.069	0.318	0.160	0.304	0.150	0.303
14	−0.5	0	0	−0.5	0.5	0	0.234	0.309	0.306	0.307	0.315	0.308	0.156	0.309	0.164	0.312
15	−0.5	0.5	0	−0.5	−0.5	0	0.158	0.401	0.156	0.493	0.158	0.498	0.155	0.304	0.159	0.310
16	−1	0	0.5	−0.5	0	0	0.113	0.233	0.158	0.306	0.069	0.158	0.156	0.309	0.067	0.157
17	−1	0	2	−0.5	0	0	0.378	0.504	0.685	0.842	0.068	0.166	0.691	0.841	0.065	0.159
18	−1	0	2	−0.5	0	0.5	0.453	0.500	0.837	0.839	0.071	0.161	0.841	0.837	0.068	0.161
19	0.5	0.5	−0.5	−0.5	0.5	−0.5	0.500	0.680	0.499	0.692	0.841	0.839	0.160	0.494	0.497	0.694
20	0.5	0.5	−0.5	−0.65	0.5	0.5	0.625	0.680	0.802	0.692	0.800	0.840	0.444	0.491	0.449	0.690

**Table 2:** Simulation results: estimated rejection probability of the designs when $x_{1}$ and $x_{2}$ are independently generated from a Bernoulli distribution with response probability 0.5. Note that “sc” denotes scenarios. The bold indicates the inflation of error rates.

sc.	($p_{A}$, $p_{B}$)	Trad	RAR	CARA1	CARA2	BaCARA
1	(0.500, 0.498)	0.056	0.046	0.055	0.061	0.040
2	(0.407, 0.398)	0.054	0.052	**0.073**	0.059	0.038
3	(0.499, 0.499)	0.051	0.046	**0.127**	0.048	0.040
4	(0.231, 0.233)	0.044	0.035	**0.075**	0.052	0.031
5	(0.501, 0.496)	0.038	0.053	**0.380**	**0.173**	0.059
6	(0.498, 0.500)	0.054	0.056	**0.105**	0.046	0.037
7	(0.658, 0.656)	0.044	0.054	**0.245**	**0.093**	0.062
8	(0.321, 0.319)	0.040	0.063	**0.094**	0.045	0.039
9	(0.344, 0.345)	0.053	0.050	**0.190**	0.064	0.051
10	(0.312, 0.499)	0.788	0.793	0.753	0.796	0.806
11	(0.235, 0.404)	0.758	0.741	0.723	0.746	0.739
12	(0.138, 0.276)	0.735	0.700	0.663	0.690	0.684
13	(0.112, 0.311)	0.942	0.935	0.941	0.927	0.922
14	(0.234, 0.309)	0.227	0.230	0.243	0.220	0.230
15	(0.158, 0.401)	0.981	0.979	0.947	0.975	0.971
16	(0.113, 0.233)	0.615	0.650	0.625	0.636	0.648
17	(0.378, 0.504)	0.416	0.392	0.516	**0.234**	0.671
18	(0.453, 0.500)	0.093	0.095	0.616	0.276	0.203
19	(0.500, 0.680)	0.752	0.748	0.889	0.775	0.815
20	(0.625, 0.680)	0.149	0.136	0.267	0.175	0.189

**Table 3:** Distribution of biomarker subgroups in each treatment arm under scenarios 5 and 7 for each design: mean (standard deviation) of the allocation probability of the treatment for each subgroup is reported. Note that “sc” denotes scenarios.

sc.	Design	Arm	Subgroups Determined by $x=(x_{1},x_{2})$			
			(1,1)	(1,0)	(0,1)	(0,0)
5	Trad	A	0.248 (0.044)	0.250 (0.042)	0.250 (0.041)	0.252 (0.041)
		B	0.250 (0.042)	0.248 (0.043)	0.252 (0.043)	0.250 (0.042)
	RAR	A	0.252 (0.043)	0.250 (0.044)	0.250 (0.043)	0.248 (0.042)
		B	0.250 (0.041)	0.248 (0.041)	0.252 (0.042)	0.251 (0.042)
	CARA1	A	0.247 (0.071)	0.252 (0.075)	0.249 (0.074)	0.252 (0.074)
		B	0.251 (0.071)	0.252 (0.074)	0.247 (0.074)	0.250 (0.074)
	CARA2	A	0.249 (0.065)	0.254 (0.056)	0.244 (0.064)	0.253 (0.058)
		B	0.241 (0.066)	0.255 (0.058)	0.247 (0.064)	0.257 (0.059)
	BaCARA	A	0.250 (0.073)	0.248 (0.071)	0.248 (0.073)	0.254 (0.073)
		B	0.248 (0.082)	0.253 (0.087)	0.247 (0.088)	0.252 (0.085)
7	Trad	A	0.249 (0.041)	0.250 (0.042)	0.251 (0.042)	0.250 (0.042)
		B	0.250 (0.044)	0.252 (0.044)	0.250 (0.043)	0.248 (0.042)
	RAR	A	0.250 (0.043)	0.250 (0.041)	0.250 (0.042)	0.250 (0.042)
		B	0.250 (0.044)	0.251 (0.043)	0.250 (0.041)	0.250 (0.042)
	CARA1	A	0.248 (0.071)	0.246 (0.073)	0.254 (0.076)	0.253 (0.081)
		B	0.243 (0.074)	0.251 (0.079)	0.247 (0.079)	0.259 (0.086)
	CARA2	A	0.245 (0.065)	0.250 (0.061)	0.249 (0.062)	0.255 (0.059)
		B	0.246 (0.065)	0.249 (0.061)	0.249 (0.062)	0.256 (0.063)
	BaCARA	A	0.244 (0.067)	0.251 (0.067)	0.250 (0.066)	0.255 (0.074)
		B	0.252 (0.079)	0.249 (0.082)	0.246 (0.081)	0.253 (0.091)

**Table 4:** Simulation results: other operating characteristics of the designs when $x_{1}$ and $x_{2}$ are independently generated from a Bernoulli distribution with response probability 0.5. Note that “sc” denotes scenarios.

sc.	($p_{A}$, $p_{B}$)	Trad	RAR	CARA1	CARA2	BaCARA
	Difference of the number of patients between A and B					
10	(0.312, 0.499)	0.136	3.038	41.114	8.278	28.990
11	(0.235, 0.404)	−0.398	2.266	41.812	7.800	28.904
12	(0.138, 0.276)	0.852	1.606	41.994	4.584	32.122
13	(0.112, 0.311)	0.064	3.082	48.808	5.256	27.088
14	(0.234, 0.309)	0.002	1.508	24.344	3.222	26.000
15	(0.158, 0.401)	0.924	2.772	45.148	7.508	19.454
16	(0.113, 0.233)	0.324	1.928	44.322	4.152	33.606
17	(0.378, 0.504)	−0.086	2.042	43.306	17.598	32.072
18	(0.453, 0.500)	−1.176	0.732	18.972	0.316	23.070
19	(0.500, 0.680)	0.652	2.796	34.500	9.792	23.950
20	(0.625, 0.680)	−0.584	0.482	16.808	4.692	17.718
	Number of failures					
10	(0.312, 0.499)	73.02	73.31	69.39	72.81	61.07
11	(0.235, 0.404)	58.47	58.58	55.66	58.47	49.02
12	(0.138, 0.276)	38.66	39.26	36.22	38.64	33.29
13	(0.112, 0.311)	34.86	34.44	30.12	34.32	25.82
14	(0.234, 0.309)	55.94	55.92	53.53	55.48	48.40
15	(0.158, 0.401)	44.10	43.29	39.69	42.94	31.20
16	(0.113, 0.233)	33.47	33.21	30.78	32.73	27.54
17	(0.378, 0.504)	87.95	87.92	80.10	89.01	72.76
18	(0.453, 0.500)	99.66	99.71	87.82	97.74	84.11
19	(0.500, 0.680)	108.95	108.80	96.76	106.50	86.62
20	(0.625, 0.680)	135.25	134.73	130.58	133.56	116.36

## Data Availability

Not applicable.

## Appendix A. Calculation of Posterior Probability

We have used Bayesian inference for personalized randomization (Section 2.4) and monitoring rule (Section 2.5). In the Bayesian probit regression model, we compute the posterior distribution instead of computing the maximum likelihood estimator of the parameter. As mentioned in Section 2.3, we assume that the regression coefficient vector $\theta ={\left(\beta^{⊤},\gamma^{⊤}\right)}^{⊤}$ follows the normal prior distributions. Given the observed data D, the posterior distribution of parameter θ is obtained to be proportional to prior distribution of parameter times the likelihood function. Then, the posterior sample is drawn from the posterior distribution using data augmentation and Gibbs sampling [43,44].

Given the accumulated data $D_{k-1}$ and *p*-dimensional covariate vector x, we compute

(A1) $$p_{k-1}\left(x\right)=\int_{R^{2p+2}}F_{A,x}\left(g_{B,x}\left(\theta \right)|D_{k-1}\right)f_{B,x}\left(\theta |D_{k-1}\right)\;d\theta$$

where $g_{B,x}\left(\theta \right)=\mathrm{Pr}\left(Y=1|G=0,x\right)=\Phi \left({\tilde{x}}^{⊤}\beta \right)$, $F_{A,x}\left(z|D_{k-1}\right)$ denotes the posterior cumulative distribution of $\mathrm{Pr}\left(Y=1|G=1,x\right)=\Phi \left({\tilde{x}}^{⊤}\beta +G{\tilde{x}}^{⊤}\gamma \right)$ evaluated at z, and $f_{B,x}\left(\theta |D_{K-1}\right)$ denotes the posterior density of $g_{B,x}\left(\theta \right)$. Then, (A1) is approximated by a Monte Carlo simulation, i.e., the posterior probability $p_{k-1}\left(x\right)$ is approximated by average of $I\left\{p_{A}\left(x\right)<p_{B}\left(x\right)\right\}=I\left\{\Phi \left({\tilde{x}}^{⊤}\beta +G{\tilde{x}}^{⊤}\gamma \right)-\Phi \left({\tilde{x}}^{⊤}\beta \right)<0\right\}$ over the posterior sample θ.

Similarly, for some value of δ, $\mathrm{Pr}(T_{k}<\delta |D_{k})$ is approximated by the average of $I(T_{k}<\delta )$ over the posterior sample based on data $D_{k}$, where $T_{k}$ is the weighted sum of $\Delta_{j}$, j=1,…,k. In both cases, the posterior samples are obtained easily by using the R package LearnBayes.
